# P-568. Comprehensive antimicrobial susceptibility testing against bacterial biodefense pathogens reveals opportunities for drug repurposing within the DoD Joint Deployment Formulary

**DOI:** 10.1093/ofid/ofaf695.783

**Published:** 2026-01-11

**Authors:** J Matthew Meinig, Stephanie Halasohoris, Bobby J Curry, Annette Gray, Jade Spencer, Jenny Chua

**Affiliations:** USAMRIID, Fort Detrick, Maryland; U.S. Army Medical Research Institute of Infectious Diseases, Fort Detrick, Maryland; U.S. Army Medical Research Institute of Infectious Diseases, Fort Detrick, Maryland; U.S. Army Medical Research Institute of Infectious Diseases, Fort Detrick, Maryland; U.S. Army Medical Research Institute of Infectious Diseases, Fort Detrick, Maryland; U.S. Army Medical Research Institute of Infectious Diseases, Fort Detrick, Maryland

## Abstract

**Background:**

The Centers for Disease Control and Prevention (CDC) Federal Select Agent Program designates several bacterial species as Tier 1 biological select agents including *Bacillus anthracis*, *Yersinia pestis*, *Francisella tularensis*, *Burkholderia mallei*, and *Burkholderia pseudomallei*. The etiological agents of anthrax, plague, tularemia, glanders, and melioidosis, respectively, these pathogens are of particular concern given the potential for deliberate misuse, mass casualties, and severe threat to public health. FDA-approved medical countermeasures against these threats are limited and the regulatory pathway remains difficult. Short of regulatory approval, off-label use and recommendations through clinical practice guidelines are alternative paths to inform clinicians of alternative therapy that can be leveraged in mass casualty or resource-limited settings. The DoD Joint Deployment Formulary (JDF) is a common, standardized list of pharmaceuticals to support the initial stage of military deployment.

**Methods:**

Here, we have tested 58 antibiotics, including all 40 antibiotics in the JDF, against 30-strain, geographically diverse panels of each pathogen at Biosafety Level (BSL)-3. Antibiotic susceptibility testing (AST) was performed in accordance with CLSI M45 guidelines using the microbroth dilution assay. As susceptibility interpretation criteria (STIC) for infections by these five pathogens are limited, FDA or EUCAST susceptibility breakpoints were assigned from other indications to guide down selection.

**Results:**

Of the tested products, 51 antibiotics were found to have MIC_90_ values for at least one pathogen in the susceptible range. To our knowledge, this is the largest effort to test antimicrobial susceptibility across these five biodefense pathogens. The production of a single-study dataset performed by the same lab under the same conditions will prove valuable to stakeholders in making pairwise comparisons not typically seen in smaller AST studies.

**Conclusion:**

Testing in this manner revealed several serendipitous findings that warrant additional investigation and point to some attractive drug repurposing targets. Prepositioning of such antibiotics will serve to strengthen military readiness and public health in the face of biological threats.

**Disclosures:**

All Authors: No reported disclosures

